# Vitrification of Donkey Sperm: Is It Better Using Permeable Cryoprotectants?

**DOI:** 10.3390/ani10091462

**Published:** 2020-08-20

**Authors:** Manuel Hidalgo, Maria Diaz-Jimenez, César Consuegra, Blasa Pereira, Jesús Dorado

**Affiliations:** Veterinary Reproduction Group, Department of Medicine and Animal Surgery, Faculty of Veterinary Medicine, University of Cordoba, 14071 Cordoba, Spain; mariadijm@gmail.com (M.D.-J.); mtc93vet@gmail.com (C.C.); blasypereiraaguilar@gmail.com (B.P.); jdorado@uco.es (J.D.)

**Keywords:** donkey, sperm, vitrification, spheres, sucrose, BSA, glycerol

## Abstract

**Simple Summary:**

Conventional donkey sperm-freezing using permeable cryoprotectants has been successfully performed, and good sperm parameters have been obtained after thawing. Unfortunately, artificial insemination of jennies with cryopreserved semen has given unsatisfactory results. Vitrification by directly dropping the sperm into the liquid nitrogen following the spheres methodology has been developed in human beings as an alternative to conventional freezing. This technique has shown to be a species-specific methodology and the concentration of cryoprotectants should be optimized in donkeys. Additionally, in this study, a permeable cryoprotectant (glycerol) has been tested for the first time for donkey sperm vitrification. According to our findings, vitrification of donkey sperm was effectively carried out using an extender supplemented with sucrose or bovine serum albumin (BSA) as non-permeable agent. When glycerol, a permeable agent, was compared to sucrose 0.1 M and BSA 5%, sperm quality significantly decreased. Therefore, donkey sperm vitrification in the absence of permeable agents obtained better results and gives a new approach to create a pattern for future studies of fertility trials.

**Abstract:**

Vitrification by direct exposure of sperm to liquid nitrogen is increasing in popularity as an alternative to conventional freezing. In this study, the effect of permeable cryoprotectant agents for donkey sperm vitrification was compared to an extender containing non-permeable cryoprotectants. First, three different concentrations of sucrose (0.1, 0.2, and 0.3 molar, M) and bovine serum albumin, BSA (1, 5, and 10%) were compared. Secondly, the concentration of non-permeable agents producing the most desirable results was compared to an extender containing glycerol as permeable agent. Vitrification was performed by dropping 30 μL of sperm suspension directly into LN2 and warming at 42 °C. Sperm motility (total, TM; and progressive, PM) and plasma membrane integrity, PMI (mean ± SEM) were statistically compared between treatments. Sucrose 0.1 M showed a significantly higher percentage of total sperm motility (21.67 ± 9.22%) than sucrose 0.2 M (14.16 ± 4.50%) and 0.3 M (8.58 ± 6.22%); and no differences were found in comparison to the control (19.71 ± 10.16%). Vitrification with sucrose 0.1 M or BSA 5% obtained similar results for TM (21.67 ± 9.22% vs. 19.93 ± 9.93%), PM (13.42 ± 6.85% vs. 12.54 ± 6.37%) and PMI (40.90 ± 13.51% vs. 37.09 ± 14.28); but both showed higher percentages than glycerol (TM = 9.71 ± 4.19%; PM = 5.47 ± 3.17%; PMI = 28.48 ± 15.55%). In conclusion, donkey sperm vitrification in spheres using non-permeable cryoprotectants exhibited better sperm motility and viability parameters after warming than sperm vitrification using extenders containing permeable cryoprotectants.

## 1. Introduction

According to the Food and Agricultural Organization, European donkey populations have diminished considerably in the last century [1]. According to the Spanish regulations, the Andalusian donkey breed is in danger of extinction, with a breeding population of 100 males and 436 females in the last update in 2020 [2]. In this sense, and considering the importance of environment and biodiversity preservation of domestic species resources, the creation of sperm banks contributes to preserve valuable genetic material from these endangered populations. Unfortunately, artificial insemination (AI) with cryopreserved donkey semen has resulted in poor fertility outcomes [3]. Different strategies have been developed to improve pregnancy rates, including the combination of different cryoprotectant agents (CPAs) [4], addition of seminal plasma to frozen-thawed donkey semen before AI [3], post-thaw centrifugation for cryoprotectant removal [5], study of the jennies endometrial response after AI [6,7], or the influence of different insemination protocols [8]. The low fertilizing capacity of cryopreserved donkey sperm has been attributed to the impact of permeable CPAs, but this hypothesis remains unclear [3,9,10].

In addition, the osmotic stress produced in the sperm cell during conventional freezing and thawing may induce structural and functional damage through the formation of ice crystals, and affects the fertilizing ability of cryopreserved sperm [11]. Vitrification is a cryopreservation method widely used for embryo, oocyte or tissue storage [12,13]. It involves the solidification of a solution, which turns into a glass-like state [14]. During vitrification, viscosity greatly increases, and intracellular or extracellular ice crystals are not formed because water does not precipitate [15]. A high concentration of permeable CPAs has been used to reach the high viscosity needed for oocyte and embryo vitrification [13]. However, this methodology has yet to be applied to the sperm cell due to its higher sensitivity to increasing concentrations of permeable CPAs [16,17]. Nevertheless, it has been demonstrated that the concentration of CPAs required for achieving vitrification is inversely related to the rate of cooling/warming. This means that if the sample is ultra-fast cooled (immersing small volume samples directly into LN_2_), high concentration of permeable CPAs are not necessary to achieve a vitrified state, therefore avoiding their toxicity [13,18]. This methodology, combined with non-permeating substances such as proteins and/or carbohydrates, has been called ’kinetic sperm vitrification’ or ‘permeable cryoprotectant-free sperm vitrification’ and has been successfully developed in human [15], dog [19], fish [20,21] wild ungulates [17,22], cats [23] and, recently, in stallions [24] and donkeys [25]. It is a simple, fast, and cost-effective method to cryopreserve sperm, even under field conditions, since a reasonably equipped laboratory is required, making it attractive for the conservation of wild or endangered species and genetically valuable animals distributed in different regions, as it usually happens in endangered donkey breeds. Taking into account that sperm vitrification has led to similar or an increase in sperm quality after warming in comparison to conventional freezing in stallion [24], it could be considered to be another alternative to improve sperm cryopreservation in donkeys.

The optimal concentration of CPAs seems to be species-specific and has been proposed as a key factor for sperm vitrification success, depending on the methodology used [24]. In a preliminary research, donkey sperm vitrified in straws showed significant higher sperm motility percentages when compared to vitrification in spheres [25]. However, only sucrose was tested as cryoprotectant agent, and a fixed concentration was employed for both methods derived from previous studies in other species. Consequently, the optimal concentration of non-permeable CPAs for donkey sperm vitrification using the spheres methodology has not been determined yet. Additionally, as previously stated, sperm vitrification has been developed using non-permeable CPAs [26] and a few attempts for sperm vitrification have been performed using a combination of permeable and non-permeable CPAs [27]. To the best of our knowledge, donkey sperm vitrification in spheres with the sole use of permeable CPAs has not been tested yet.

Therefore, the present study was designed to examine the effectiveness of different concentrations of sucrose and bovine serum albumin for donkey sperm vitrification in spheres, in comparison to a vitrification extender containing permeable CPAs by examining the post-thaw quality in vitro.

## 2. Materials and Methods

### 2.1. Animals, Semen Collection, and Processing

All procedures were approved by the Ethical Committee for Animal Experimentation of the University of Cordoba, in compliance with the Regional Government of Andalusia (project no. 31/08/2017/105) and the Spanish law for animal welfare and experimentation (RD 53/2013).

The jackasses were housed in individual paddocks at “Centro Rural Malpica” (Palma del Rio, Cordoba, Spain). They were fed with teff hay, oat grains and water “ad libitum”. Semen was collected from four adult, healthy and fertile Andalusian donkeys using a Missouri artificial vagina (Minitüb, Tiefenbach, Germany) in the presence of a jenny in estrus. Semen was collected one or twice a week per donkey until a total of 16 ejaculates was completed (four ejaculates per donkey). Immediately after collection, volume (mL) and sperm concentration (×10^6^ spermatozoa/mL) were measured in each gel-free semen sample using a graduated collector and a sperm photometer (Spermacue^®^, Minitüb, Tiefenbach, Germany) respectively. Sperm motility and viability parameters were evaluated as described below. Thereafter, sperm was extended 1:1 (*v/v*) with INRA96 (IMV Technologies, France) and centrifuged at 400× *g* for 7 min to remove seminal plasma. Sperm pellets were then re-extended in each vitrification media (see experimental design) to reach a final concentration of 200 × 10^6^ spermatozoa/mL. Sperm suspensions were maintained at room temperature for 10 min and slowly cooled for 1 h at 5 °C into a sperm container (Equitainer, Hamilton Research, Inc. Ipswich, Massachusetts, USA) before the vitrification procedure was performed.

### 2.2. Vitrification and Warming

**Figure 1:**
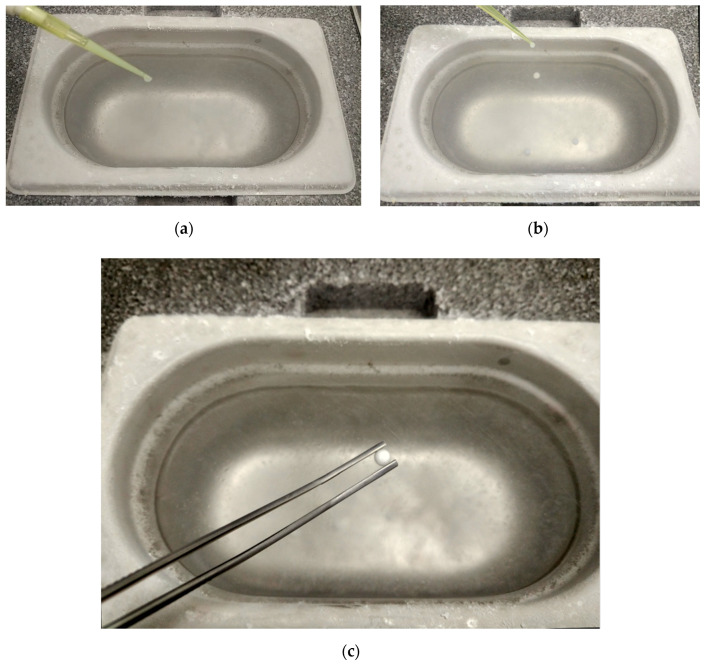
Images of the spermatozoa vitrification procedure, (**a**,**b**) 30 μL of spermatozoa suspension dropped into LN_2_ using a micropipette, (**c**) sphere formed after sperm vitrification.

Sperm vitrification was carried out following the methodology previously described [24,25]. Briefly, a styrofoam box was loaded with LN_2_ and five 30 µL drops from each treatment were plunged directly into the LN_2_. A micropipette held at an angle of about 45° and a distance of 10 cm from the surface was used (Figure 1a,b). After contact with the LN_2_ a sphere is immediately formed (Figure 1c). Spheres were then packaged into 1.8 mL cryotubes and stored LN_2_ tanks. The warming procedure was performed by introducing the spheres one by one into two milliliters of extender (INRA-96) previously warmed to 42 °C. A gentle vortexing for a few seconds of each sample was done before centrifugation as described above. Sperm pellets were re-extended with INRA-96 after supernatant removal, to reach final concentration of 25 × 10^6^ spermatozoa/mL for sperm evaluation. Sperm motility was objectively evaluated by using the Sperm Class Analyzer (SCA v.5.4.0, Microptic S.L., Barcelona, Spain) as previously described [4]. The following kinetic parameters were calculated by the system: total (TM, %) and progressive motility (PM, %); curvilinear (VCL, µm/s), straight line (VSL, µm/s) and average path velocities (VAP, µm/s), linearity (LIN, VSL/VCL × 100), straightness rate (STR, VSL/VAP × 100), wobble (WOB, VAP/VCL × 100), lateral head displacement amplitude (ALH, µm) and beat cross frequency (BCF, Hz). Sperm membrane integrity was assessed using Vital-Test commercial kit (Halotech DNA S.L., Madrid, Spain) for sperm staining following the manufacturer´s instructions. In brief, an aliquot of 10 μL of diluted semen was mixed with 1 μL propidium iodide stock solution and 1 μL of acridine orange stock solution and evaluated under epifluorescence microscopy. At least 200 spermatozoa were counted, and sperm with intact plasma membrane was recorded (PMI, %).

### 2.3. Experimental Design

#### 2.3.1. Experiment 1. Vitrification of Donkey Sperm Using Different Concentrations of Sucrose

Sucrose (Sigma-Aldrich Corp., St. Louis, MI, USA) was added to a control base extender (Control) commonly used for horse sperm. This base extender contains egg yolk and antibiotics (Gent, Minitüb, Tiefenbach, Germany). Powder sucrose was weighted with a precision balance and mixed with the control extender by vortexing. Three concentrations of sucrose (mol/L, M) were compared: 0.1 M, 0.2 M and 0.3 M; each extender was then added to the sperm pellets and vitrified as described before. Post-warming sperm parameters were recorded and compared between treatments.

#### 2.3.2. Experiment 2. Vitrification of Donkey Sperm Using Different Concentrations of Bovine Serum Albumin

Bovine serum albumin (BSA, Sigma-Aldrich, Sant Louis, MI, USA) was weighted and added to the same control base extender as described before at the following concentrations: 1%, 5% and 10%. Each sample was then vitrified, and post-warming analysis was carried out as previously described, comparing the results obtained among treatments.

#### 2.3.3. Experiment 3. Comparison Between Permeable and Non-Permeable CPAs for Donkey Sperm Vitrification

Having identified the best concentration of non-permeable CPAs for donkey sperm vitrification in spheres, a commercial extender for stallion sperm-freezing containing permeable CPAs, in particular glycerol (Gent B, Minitüb, Tiefenbach, Germany), was compared for sperm vitrification. Post-warming sperm parameters were assessed for each treatment as described before.

### 2.4. Statistical Analysis

Results were analyzed using the Statistical Analysis Systems software (SAS version 9.0; SAS Institute Inc, Cary, NC, USA). All data was first tested for normality of the data distribution and homogeneity of variances using the Kolmogorov–Smirnov and Levene test, respectively. The sperm parameters assessed were compared between treatments using a repeated measured general lineal model (GLM). Animals and ejaculates were considered to be random factors. The Post-Hoc HSD Tukey test was used to compare differences between mean values. Results were expressed as mean ± standard deviation of the mean (SD). The level of significance was set at *p* < 0.05.

## 3. Results

Mean average parameters of ejaculates used in this study were as follows: gel-free volume of 112 ± 41 mL, sperm concentration of 191 ± 68 × 10^6^ spermatozoa/mL, total motility 79.7 ± 12.8%, progressive motility 64.7 ± 15.4% and sperm with intact plasma membrane 58.7 ± 15.7%.

### 3.1. Experiment 1. Vitrification of Donkey Sperm Using Different Concentrations of Sucrose

Vitrification in spheres using a sucrose concentration of 0.1 M resulted in the greatest values (*p* < 0.05) for all the sperm motility variables assessed in comparison to the other sucrose concentrations (Table 1). There were no significant differences (*p* > 0.05) between control and sucrose treatments for the assessment of plasma membrane integrity. There were no differences between sucrose 0.1 M and the control for total (21.67 ± 9.22 vs. 19.71 ± 10.16) and progressive (13.42 ± 6.85 vs. 12.34 ± 8.13) sperm motility, respectively; however, sucrose 0.1 M showed a tendency to obtain higher values of sperm motility percentages.

### 3.2. Experiment 2. Vitrification of Donkey Sperm Using Different Concentrations of Bovine Serum Albumin

The addition of different concentrations of BSA to the vitrification extender resulted in no significant differences (*p* > 0.05) in any of the sperm parameters assessed when compared to control samples (Table 2).

### 3.3. Experiment 3. Comparison Between Permeable and Non-Permeable CPAs for Donkey Sperm Vitrification

The sole use of glycerol for sperm vitrification in spheres decreased (*p* < 0.05) TM (9.71 ± 4.19%), PM (5.47 ± 3.17%) and PMI (28.48 ± 15.55%) compared to vitrification using sucrose 0.1 M and BSA 5% (Table 3). No significant differences (*p* > 0.05) were found in the remaining sperm parameters assessed.

## 4. Discussion

Permeable CPAs have been conventionally employed for the cryopreservation of donkey sperm samples by slow freezing in donkey sperm, and by vitrification in spheres in other species, but they had not been employed for vitrification in donkeys yet. Non-permeable CPAs have been successfully used for sperm vitrification in different animal species [15,19,24,25,28]. It has been pointed out that the optimal concentration of non-permeable cryoprotectants varies between vitrification techniques. In this sense, studies comparing different concentrations of non-permeable cryoprotectants for sperm vitrification using the spheres technique in donkey sperm are so far lacking in the scientific literature.

In the first experiment of the present study, the addition of sucrose 0.1 M to the extender had a positive effect on sperm motility percentages in comparison to the control without sucrose. Sucrose concentrations were selected taking into consideration other reports of sperm vitrification in spheres in mammals [12,17,19,23,24,25,29], in which various amounts of sucrose between 0.02 M to 0.5 M were added. According to the results obtained in this study, the lowest sucrose concentration (0.1 M) resulted in the greatest values for sperm motility parameters in comparison to higher concentrations of sucrose (0.2 M and 0.3 M). These results are in agreement with previous reports in which different sucrose concentrations were compared, and higher sucrose concentrations (0.3 M and 0.5 M) resulted in significantly lower values for sperm motility in comparison to 0.1 M in wild goat [17] and sheep [22]. Similarly, sucrose 0.1 M has also been successfully used for sperm vitrification in spheres in 14 different wild endangered species [30] and in mouflon and fallow deer [31]. However, previous research of sperm vitrification using the spheres method have shown sucrose requirements to be slightly higher than 0.1 M in other species. In this regard, concentrations of 0.2 M [32] and 0.25 M [29,33] have been successfully employed in studies performed in human; 0.125 M in fish [20]; 0.2 M in cat [23], and 0.25 M in dog [19]. Interestingly, though, despite the phytogenic proximity between horse and donkey species [24], sucrose requirements for vitrification of stallion sperm showed to be much lower than that of in donkeys. It has been reported the upper limit of sucrose concentration to be 0.02 M in stallions [24], which is far lower than those used in the present study.

Our results once again reaffirm the previously described differences of cryoprotectant requirements between species [22,24]. A reasonable explanation for the diverse responses to vitrification extender is that species have different sperm cryosurvival, as described by other authors [12,27]; it seemed to be species-specific, which in turn may be a consequence of the cryostability and properties of the sperm cell: sperm size, water content, membrane fluidity, osmotic content and/or internal compaction [30,31,34]. In agreement with previous reports in other species [17,28], in the current study no differences were found between the different sucrose concentrations and the control extender regarding plasma membrane integrity.

Serum albumin has been traditionally added to the vitrification extender for sperm cryopreservation in several species. Thus, human serum albumin has been employed at 1% in human [29]; and BSA at 0.5% in rabbit [12]; 1% in equine [35], dog [19], fish [28], donkey [36] and wild goat [17]; and 2% in ram [37]. This molecule has shown to reduce oxidative stress [38] and to protect the sperm membrane against cryodamage, although the exact mechanism is still not clear [39]. Few studies have, however, tested different concentrations for sperm vitrification to determine the most adequate [24]. Moreover, to the best of the authors knowledge, the effect of BSA for sperm vitrification has always been examined in combination with sucrose and other cryoprotectants, but not by itself. Therefore, we aimed to test if the sole use of BSA could increase sperm quality after vitrification. Surprisingly, no differences among BSA concentrations nor with the control extender were found in any of the parameters assessed. However, the concentration of BSA 5% showed a tendency to obtain higher motility results.

In the last experiment, and considering that the use of 0.1 M of sucrose in the extender highly improved motility parameters, and BSA 5% also showed a tendency to improve motility results, both extenders were compared with an extender with a permeable CPA for donkey sperm vitrification. Glycerol was selected as the permeable agent because it has been widely and successfully employed for donkey sperm cryopreservation in previous studies [3,4,40,41,42], and could be considered to be a starting point to study the impact of permeable agents in donkey sperm vitrification. Glycerol has been previously included in vitrification extenders following the spheres or straws method, with positive results in sperm quality parameters after warming in several species such as ram [16] and sea bream [43]; dimethyl sulfoxide in salmon [21], and a combination of ethylene glycol and dimethyl sulfoxide in goat [44]. According to our results, the addition of glycerol to the vitrification extender significantly reduced sperm motility and plasma membrane integrity after warming; however, similar results were obtained when sperm vitrification was performed using either sucrose 0.1 M or BSA 5% as non-permeable agents. These results agree with previous reports in wild sheep [22], in which glycerol addition to the vitrification extender decreased sperm motility and plasma membrane integrity compared to the sole use of non-permeable CPAs. Nonetheless, other authors reported no motile or viable boar sperm after vitrification with only sucrose, neither in combination with permeable agents [27]. Sperm vitrification in spheres using only non-permeable agents have also been problematic in other species, obtaining few motile or viable sperm after warming in rabbit [12] and ram [37]. Interestingly, in a previous study performed in donkeys [42] in which authors compared between slow freezing using only non-permeable agents and the same freezing protocol but containing glycerol, no differences were found for sperm motility and DNA integrity. Therefore, it seems that glycerol may protect donkey sperm during slow freezing but no during vitrification. As mentioned before, these differences among studies can be explained in part by the cryopreservation method employed, an inadequate concentration and/or type of non-permeable agent [12], sperm cryosurvival regarding the species [27], as well as the lack of equilibration temperature, which have shown to be essential for sperm vitrification in spheres [24].

## 5. Conclusions

The present study showed that donkey sperm could not be vitrified in small volumes (spheres) using only glycerol as permeable CPAs. Vitrification using non-permeable CPAs (sucrose 0.1 M and BSA 5%) enhanced sperm motility and viability after warming. Further studies will concentrate on evaluating combinations of permeable and non-permeable CPAs for donkey sperm vitrification, assessing a wider range of sperm parameters after warming.

## Figures and Tables

**Table 1:** Vitrification of donkey semen samples (*n* = 16) using different concentrations of sucrose.

Sperm Parameters	Vitrification Extender				*p*-Values
	Control	Sucrose 0.1 M	Sucrose 0.2 M	Sucrose 0.3 M	
TM (%)	19.71 ± 10.16 ^a,b^	21.67 ± 9.22 ^a^	14.16 ± 4.50 ^b^	8.58 ± 6.22 ^c^	<0.001
PM (%)	12.34 ± 8.13 ^a^	13.42 ± 6.85 ^a^	7.69 ± 3.01 ^b^	3.53 ± 4.42 ^b^	<0.001
PMI (%)	33.66 ± 14.84	40.90 ± 13.50	39.67 ± 13.12	38.62 ± 10.32	>0.05
VCL (µm/s)	77.04 ± 19.70 ^a^	82.16 ± 13.31 ^a^	65.07 ± 13.04 ^b^	44.69 ± 16.98 ^c^	<0.001
VSL (µm/s)	62.52 ± 19.12 ^a^	66.91 ± 12.48 ^a^	50.42 ± 13.80 ^b^	32.61 ± 15.43 ^c^	<0.001
VAP (µm/s)	67.23 ± 19.51 ^a,b^	71.96 ± 12.93 ^a^	56.46 ± 11.74 ^b^	36.75 ± 16.58 ^c^	<0.001
ALH (µm)	2.34 ± 0.29 ^a^	2.46 ± 0.43 ^a^	2.04 ± 0.56 ^b^	1.78 ± 0.67 ^b^	<0.001
LIN (%)	80.02 ± 5.69 ^a^	81.16 ± 6.19 ^a^	76.52 ± 11.01 ^a,b^	68.13 ± 17.85 ^b^	<0.05
STR (%)	92.74 ± 2.80 ^a^	92.84 ± 2.85 ^a^	88.08 ± 11.32 ^a,b^	84.44 ± 17.97 ^b^	<0.05
WOB (%)	86.40 ± 4.32 ^a^	87.34 ± 4.64 ^a^	86.70 ± 3.79 ^a^	78.35 ± 13.85 ^b^	<0.05
BCF (Hz)	9.65 ± 0.82 ^a^	9.64 ± 0.66 ^a^	8.60 ± 1.79 ^a,b^	7.68 ± 2.90 ^b^	<0.001

Different letters (^a–c^) in the same row indicate significant differences. TM, total motility; PM, progressive motility; PMI, plasma membrane integrity; VCL, curvilinear velocity; VSL, straight line velocity; VAP, average path velocity; ALH, amplitude of lateral head displacement; LIN, linearity; STR, straightness; WOB, wobble; BCF, beat cross frequency; Control, control extender without sucrose. Values are expressed as mean ± standard deviation of the mean.

**Table 2:** Vitrification of donkey semen samples (*n* = 16) using different concentrations of bovine serum albumin (BSA).

Sperm Parameters	Vitrification Extender				*p*-Values
	Control	BSA-1%	BSA-5%	BSA-10%	
**TM (%)**	19.71 ± 10.16	19.51 ± 9.67	19.93 ± 8.93	15.52 ± 6.37	>0.05
**PM (%)**	12.34 ± 8.13	12.58 ± 7.65	12.54 ± 6.37	9.24 ± 4.39	>0.05
**PMI (%)**	33.66 ± 14.84	36.37 ± 11.36	37.09 ± 14.28	35.49 ± 13.28	>0.05
**VCL (µm/s)**	77.04 ± 19.70	83.74 ± 15.80	83.27 ± 16.31	79.69 ± 16.87	>0.05
**VSL (µm/s)**	62.52 ± 19.12	68.74 ± 14.78	68.19 ± 15.88	64.57 ± 17.07	>0.05
**VAP (µm/s)**	67.23 ± 19.51	74.54 ± 15.59	73.86 ± 16.43	69.91 ± 17.06	>0.05
**ALH (µm)**	2.34 ± 0.29	2.31 ± 0.36	2.46 ± 0.22	2.49 ± 0.31	>0.05
**LIN (%)**	80.02 ± 5.69	81.72 ± 4.40	81.09 ± 5.06	80.14 ± 5.74	>0.05
**STR (%)**	92.74 ± 2.77	92.12 ± 2.73	92.08 ± 2.48	91.96 ± 3.16	>0.05
**WOB (%)**	86.40 ± 4.31	88.67 ± 3.28	88.04 ± 3.95	87.06 ± 4.13	>0.05
**BCF (Hz)**	9.65 ± 0.82	9.67 ± 0.62	14.82 ± 21.65	15.16 ± 21.58	>0.05

TM, total motility; PM, progressive motility; PMI, plasma membrane integrity; VCL, curvilinear velocity; VSL, straight line velocity; VAP, average path velocity; ALH, amplitude of lateral head displacement; LIN, linearity; STR, straightness; WOB, wobble; BCF, beat cross frequency; Control, control extender without BSA; Values are expressed as mean ± standard deviation of the mean.

**Table 3:** Comparison between permeable (glycerol) and non-permeable (sucrose and BSA) cryoprotectants for donkey sperm vitrification (*n* = 16).

Sperm Parameters	Vitrification Extender			*p*-Values
	Glycerol	Sucrose 0.1 M	BSA-5%	
TM (%)	9.71 ± 4.19 ^b^	21.67 ± 9.22 ^a^	19.93 ± 8.93 ^a^	<0.001
PM (%)	5.47 ± 3.17 ^b^	13.42 ± 6.85 ^a^	12.54 ± 6.37 ^a^	<0.001
PMI (%)	28.48 ± 15.55 ^b^	40.90 ± 13.51 ^a^	37.09 ± 14.28 ^a^	<0.05
VCL (µm/s)	80.72 ± 17.14	82.16 ± 13.31	83.27 ± 16.31	>0.05
VSL (µm/s)	68.09 ± 18.06	66.91 ± 12.48	68.19 ± 15.88	>0.05
VAP (µm/s)	71.97 ± 17.55	71.96 ± 12.93	13.86 ± 16.43	>0.05
ALH (µm)	2.32 ± 0.54	2.46 ± 0.43	2.46 ± 0.22	>0.05
LIN (%)	83.04 ± 6.19	81.16 ± 6.19	81.09 ± 5.06	>0.05
STR (%)	93.68 ± 3.26	92.84 ± 2.85	92.08 ± 2.48	>0.05
WOB (%)	88.56 ± 4.26	87.34 ± 4.64	88.04 ± 3.95	>0.05
BCF (Hz)	9.47 ± 1.36	9.64 ± 0.66	14.82 ± 21.65	>0.05

Different letters (^a,b^) in the same row indicate significant differences. TM, total motility; PM, progressive motility; PMI, plasma membrane integrity; VCL, curvilinear velocity; VSL, straight line velocity; VAP, average path velocity; ALH, amplitude of lateral head displacement; LIN, linearity; STR, straightness; WOB, wobble; BCF, beat cross frequency; Values are expressed as mean ± standard deviation of the mean.
